# Looking back and looking forward: contributions of electron microscopy to the structural cell biology of gametes and fertilization

**DOI:** 10.1098/rsob.200186

**Published:** 2020-09-16

**Authors:** Ravi Teja Ravi, Miguel Ricardo Leung, Tzviya Zeev-Ben-Mordehai

**Affiliations:** 1 Cryo-Electron Microscopy, Bijvoet Centre for Biomolecular Research, Utrecht University, 3584CH Utrecht, The Netherlands; 2 Division of Structural Biology, Wellcome Centre for Human Genetics, The University of Oxford, Oxford OX3 7BN, UK

**Keywords:** electron microscopy, cryo-electron tomography, cryo-focused ion beam milling, sperm, egg, fertilization

## Abstract

Mammalian gametes—the sperm and the egg—represent opposite extremes of cellular organization and scale. Studying the ultrastructure of gametes is crucial to understanding their interactions, and how to manipulate them in order to either encourage or prevent their union. Here, we survey the prominent electron microscopy (EM) techniques, with an emphasis on considerations for applying them to study mammalian gametes. We review how conventional EM has provided significant insight into gamete ultrastructure, but also how the harsh sample preparation methods required preclude understanding at a truly molecular level. We present recent advancements in cryo-electron tomography that provide an opportunity to image cells in a near-native state and at unprecedented levels of detail. New and emerging cellular EM techniques are poised to rekindle exploration of fundamental questions in mammalian reproduction, especially phenomena that involve complex membrane remodelling and protein reorganization. These methods will also allow novel lines of enquiry into problems of practical significance, such as investigating unexplained causes of human infertility and improving assisted reproductive technologies for biodiversity conservation.

## 1. Introduction

Mammalian gametes represent extremes of cellular organization. The male gamete—the sperm—has lost most of its cytoplasm and many of the organelles present in its somatic counterparts, thus becoming a small, streamlined cell. The female gamete—the egg—accumulates cytoplasm in preparation for embryonic development, thus becoming one of the largest cell types. Because cellular function is inextricably linked to subcellular organization, knowledge of gamete ultrastructure is vital for understanding how these cells function and interact.

Since the invention of the light microscope in the 1600s, microscopy has become one of the most powerful methods for the cell biologist. Microscopy occupies a unique position in the pantheon of cell biology techniques because it allows direct observation of biological phenomena. Indeed, the history of gamete biology is intimately tied to the development of microscopy. It was, after all, van Leeuwenhoek himself who first described sperm [1]. The light microscope also played a central role in the first descriptions of oocytes [2] and provided direct evidence that fertilization involves gamete fusion [3]. With recent developments such as expansion microscopy and super-resolution light microscopy, specific proteins can now be localized in cells to within tens of nanometres [4–6]. However, this often comes at the expense of capturing cellular complexity in its entirety, as only specifically labelled proteins are imaged.

Electron microscopy (EM), on the other hand, can yield complete snapshots of the cellular interior at nanometre resolution without the need to label specific proteins [7,8]. Following the invention of the electron microscope in 1931 and the first attempts to study biological samples by transmission electron microscopy (TEM) in 1934 [9], the technique made it possible to image cells and tissues at much higher resolutions than light microscopy. Gamete structure was studied with EM from the early days of the method [10–13]. EM provided valuable insights into the ultrastructure of gametes and their interactions, which in combination with advancements in gamete cell biology and biochemistry led ultimately to successful *in vitro* fertilization (IVF) and other assisted reproductive technologies (ART) [14,15].

Cellular EM does not come without its challenges, however. Because electrons scatter strongly in air, the electron microscope needs to be operated under vacuum—a particularly hostile environment for cells, which are composed mostly of water. Furthermore, electrons used for imaging are accelerated to very high energies that are damaging to biological samples, which are inherently radiation-sensitive. Over the years, many approaches were developed to protect biological samples within the EM. Classical or conventional approaches involve sample fixation, dehydration, resin embedment and heavy metal staining [16].

Although conventional EM has yielded immense insight into a myriad of cellular processes, the harsh sample preparation methods limit achievable resolution and, moreover, can alter cellular structure and introduce artefacts [17,18]. Excitingly, the past few years have seen a methodological revolution in cellular EM. At the forefront of this revolution are methods based on rapid vitrification of samples and subsequent imaging at cryogenic temperature, collectively referred to as cryo-electron microscopy (cryo-EM). Cryo-EM addresses many of the limitations of conventional EM, though it also faces a unique set of challenges.

In this review, we survey the prominent EM techniques that shaped our knowledge of gamete cell biology, with an emphasis on mammalian systems. We cover conventional EM and cryo-EM, discussing specific considerations for applying them to questions of gamete and reproductive biology. We discuss challenges and limitations for cellular cryo-EM as well as current efforts to address them.

## 2. EM modalities and conventional EM sample preparation

There are two main EM imaging modalities, and the choice of the appropriate method depends ultimately on the biological question. In scanning electron microscopy (SEM), a focused beam of electrons is rastered across the sample. Electrons that bounce back from the surface are used to generate an image that provides topographical information on the sample [19]. In TEM, images are formed from electrons that are passed through and interact with the sample. Unlike in SEM, TEM provides information on the cell interior as well as the cell periphery. However, because electrons need to penetrate the sample in order to generate an image, TEM has a strict thickness limit. Consequently, samples thicker than approximately 1 µm (for an electron at an accelerating voltage of 300 kV) [20] are not suitable for high-resolution TEM. On the other hand, SEM is relatively flexible in terms of sample size and can be applied to samples on the order of centimetres.

Each imaging modality has dedicated sample preparation approaches, but a common feature for both is the need to protect samples from high vacuum and from radiation damage. Thus, conventional sample preparation steps typically include fixation, careful dehydration to prevent sample collapse, and staining to increase contrast. Fixation is often based on the use of chemicals such as aldehydes and osmium tetroxide to cross-link proteins. In the dehydration step, organic solvents are used to replace cellular water.

In SEM, samples must be dried carefully in order to prevent structural collapse due to surface tension. This is often achieved through a method known as critical point drying, which is based on replacing biological water with liquid CO_2_ and subsequently raising pressure and temperature to a point where the liquid-to-gas phase change can occur while keeping surface tension close to zero. The sample is then covered with a conductive coating to reduce the surface charging effects caused by electron irradiation [19].

For TEM, subsequent steps aim towards preparing sections thin enough for imaging. This involves embedding the sample in epoxy resins that are then polymerized into a solid block. The block is then cut with a diamond knife to obtain thin sections of desired thickness. These sections are then mounted on EM grids and are stained with heavy metals like uranium or lead salts to enhance contrast [21]. If protein localization is desired, antibodies coupled to gold particles can be used before embedding, following, for example, the Tokuyasu technique for immunogold labelling [22].

Fixation and dehydration steps are notorious for introducing artefacts, but over the years approaches aimed at alleviating some of these effects have been developed. These methods are based on fixation and dehydration at low temperatures. In freeze-substitution, ice in frozen cells is replaced by an organic solvent at temperatures below that at which secondary ice crystals can grow (below −70°C) [23]. In freeze-fracture and etching, a frozen sample is broken open to reveal internal structures, and further sublimation of surface ice under vacuum exposes details of the fractured face that were originally hidden (etching) [24,25]. Surface replica/metal shadowing involves deposition of a metal/carbon mix under high vacuum and dissolution of the biological material that create a metal replica of the sample [26].

## 3. Conventional EM has contributed greatly to our understanding of gamete biology and fertilisation

**Figure 1.**
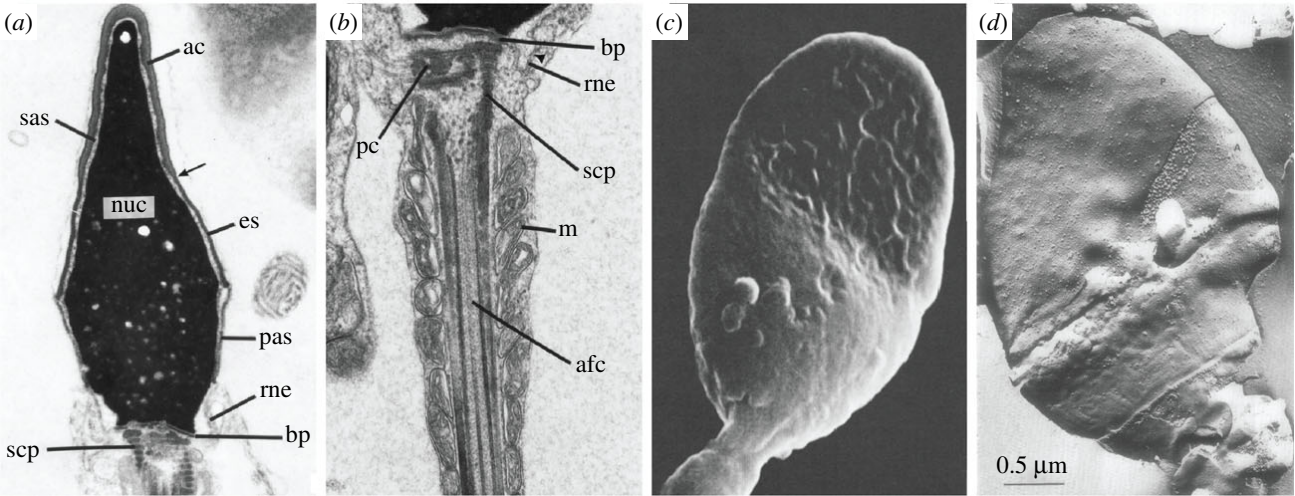
Conventional EM of mammalian spermatozoa. (*a*) TEM micrograph of a thin section of human sperm head (×30 500): ac, acrosomal cap region; sas, subacrosomal space; nuc, nucleus; es, equatorial segment; pas, postacrosomal sheath; rne, redundant nuclear envelope; bp, basal plate; scp, striated connecting piece [27]. (*b*) TEM micrograph of a longitudinal thin section through the neck and proximal tail regions of a human spermatozoon (×64 000): bp, basal plate; rne, redundant nuclear envelope (nuclear pores indicated by the triangle); pc, proximal centriole; scp, striated connecting piece; m, mitochondria; afc, axial filament complex (comprising the axoneme and dense fibres) [27]. (*c*) SEM micrograph of a human spermatozoon, showing the surface morphologies in the anterior and posterior regions of the head (×15 000) [28]. (*d*) Freeze etching electron micrograph of a human spermatozoon head, depicting a face of the acrosomal membrane beneath a portion of the overlying plasma membrane (×50 000) [29].

**Figure 2.**
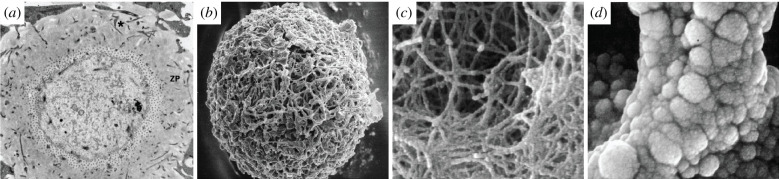
Conventional EM of mammalian oocytes. (*a*) Thin-section TEM micrograph through the cortex of a mouse oocyte, showing the extent of the zona pellucida (ZP) and numerous transzonal projections (*), which are thin cytoplasmic extensions of the cumulus granulosa cells connecting them to the oocyte, penetrating through it [30]. (*b*) SEM micrograph of a mature human oocyte, showing the porous nature of the outer surface of the ZP (×1200) [31]. (*c*) SEM micrograph of the outer surface of the ZP of a mature human ooctye at a higher magnification, showing the filamentous-like arrangement of globule-bearing structures (×50 000) [31]. (*d*) SEM micrograph at a very high magnification of an unfertilized mouse oocyte, showing a branch of the filamentous structure of the ZP (×50 000) [32].

**Figure 3.**
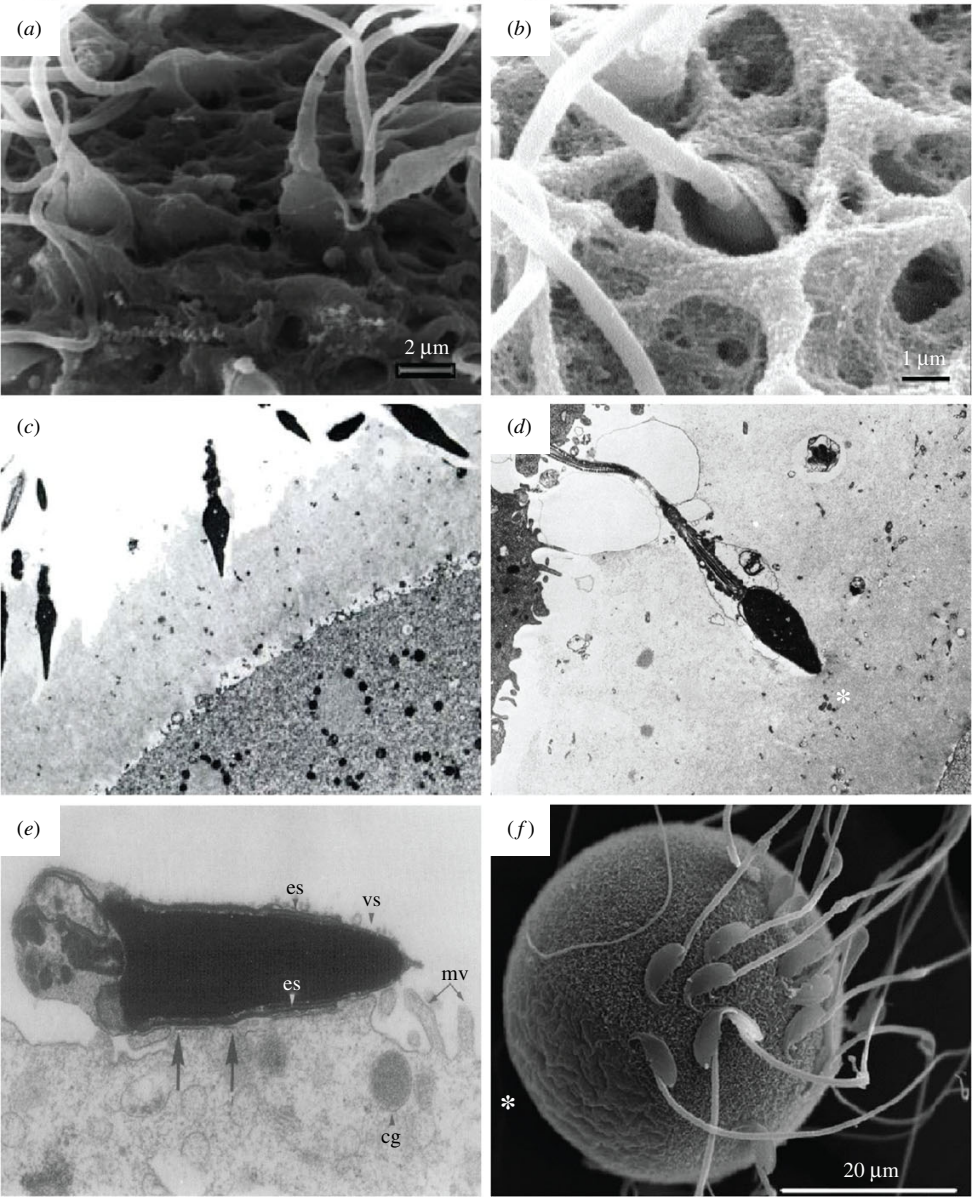
Conventional EM of mammalian sperm–oocyte interactions. (*a*) SEM micrograph of a mature human oocyte, showing the vertical binding of the sperm with a penetration of the apical tip of the sperm head into the ZP [33]. (*b*) SEM micrograph of a human sperm–oocyte interaction, showing the vertical binding of a sperm head vanishing into the the ZP [14]. (*c*) TEM micrograph of human sperm–oocyte interaction *in vitro*, showing acrosome-reacted sperm invading the ZP of a polyspermic ovum at different angles (×3330) [34]. (*d*) TEM micrograph of human sperm–oocyte interaction *in vitro*, showing an acrosome-reacted sperm that has penetrated about half the thickness of the ZP, just blocked outside the inner surface of the ZP (*) which is denser and more compact than the outer surface, thus depicting the block to polyspermy (×4330) [34]. (*e*) TEM micrograph showing the fusion of a tubal mouse egg incubated with capacitated epididymal sperm for 60 min. It shows the fusion of the sperm at the postacrosomal cap of the equatorial segment (es); cg, cortical granules; mv, microvilli; vs, vesiculated plasma and outer acrosomal membranes (×31 200) [35]. (*f*) SEM micrograph of a wild-type mouse egg incubated with sperm for 25 min, clearly showing sperm are not bound to microvilli-free region (*) [36].

Much of our current knowledge of gamete structure comes from conventional EM studies (figures 1–3). These efforts have revealed that, beyond their characteristic size and shape, gametes are also specialized at the subcellular level, with organelles that are often highly modified relative to their somatic counterparts.

During sperm development and maturation, sperm lose most of their cytoplasm and a number of their organelles. Conventional TEM was instrumental both in the study of spermatogenesis (recently reviewed in [37]) and of mature sperm (recently reviewed in [38]), revealing that in mature sperm, the structures that do remain adopt specialized sperm-specific configurations. For instance, conventional EM demonstrated that mammalian sperm mitochondria are arranged in a tight spiral around the flagellar axoneme [39–41], and that the mammalian sperm axoneme is surrounded by a plethora of accessory structures that dwarf the axoneme proper [13] (figure 1*b*). These characteristics are general features of mammalian sperm, but details such as the number of mitochondria in the midpiece and the sizes of the accessory elements vary across species (reviewed in [12,13]).

Conventional TEM was instrumental in the prognosis of male fertility by identifying teratozoospermia [42,43]. Likewise, SEM has been applied extensively to assess the detailed surface morphologies of normal and abnormal spermatozoa of humans and other mammalian species [28,44,45]. SEM can also be used as a tool to evaluate and score morphological abnormalities of spermatozoa in infertile men [46]. Motility is pivotal to sperm function and a combination of SEM with TEM of several mammalian species revealed potential relationships between sperm motility and mitochondrial functions [47].

However, despite decades of EM work, new structures are still being discovered in the deceptively simple sperm cell. For instance, it was only recently recognized that in non-rodent mammals, the atypical distal centriole at the base of the flagellum is in fact a functional centriole [4]. Previously, the distal centriole was thought to degenerate during spermiogenesis, leaving mature sperm with a single functional centriole, the proximal centriole. Given that zygotic centrioles are paternally inherited in non-rodent mammals, the origin of the second centriole was a mystery. The discovery that the distal centriole is functional yet structurally atypical is an elegant example of how explorations of cell structure can still help resolve important questions.

Freshly ejaculated mammalian sperm are unable to fertilize oocytes; they must first undergo a ‘capacitation’ phase culminating in the acrosome reaction to render them fusion-competent [48]. Both SEM and TEM helped define the physiological and morphological changes that sperm undergo during capacitation and the acrosome reaction. TEM revealed that the acrosome reaction is a dramatic membrane remodelling event culminating in vesiculation of the acrosome [49,50]. Freeze-fracture/etching revealed fine surface features of the sperm head (figure 1*d*) [29], and showed that sperm surface proteins are re-organized and re-distributed during the acrosome reaction to facilitate interaction and fusion with the egg [51].

Mammalian oocytes occupy the opposite end of the cellular size scale, growing to become one of the largest cell types (e.g. approx. 100 µm for human oocytes and approx. 150 µm for bovine oocytes). Furthermore, they are surrounded by a thick extracellular coat of glycoproteins known as the zona pellucida (ZP), which is in turn surrounded by a layer of granulosa cells. Together, these properties make them difficult samples for TEM. Nevertheless, thin serial section and freeze-substitution TEM (figure 2*a*) were instrumental in describing how mammalian oocyte organization changes during growth and maturation in the adult ovarian follicle [35,52–55]. Conventional EM also revealed that mouse oocytes are polar [35]. Over most of its surface, the oocyte plasma membrane (called the oolemma) is covered with microvilli, with numerous underlying cortical granules; however, the region overlying the meiotic spindle is much smoother and is almost devoid of cortical granules.

The ZP extracellular matrix has also been characterized extensively by conventional EM. It was shown to be composed of long interconnected filaments forming an elastic porous coat (figure 2*b–d*) [32,33,56–58]. Each filament resembles ‘beads-on-a-string’ with consistent repeats [58]. The ZP plays important roles both during fertilization, where it acts to block polyspermy, and during embryo development, to protect the growing embryo. EM showed that structural changes in the ZP are associated with these changing roles, revealing remarkable architectural plasticity in this large extracellular scaffold [31,33,59,60].

Conventional EM was instrumental in visualizing sperm–egg interactions leading to fertilization (figure 3). Mammalian sperm can attach to and penetrate the ZP at various angles (figure 3*a*–*d*) [14,34], but seem to only interact tangentially with the oolemma via a specialized cell surface domain known as the equatorial segment (figure 3*e,f*). Egg microvilli are actively involved in fusion with the equatorial segment [61]. EM-based immunogold labelling localized CD9 and Juno, two proteins essential for fertilization, to the microvilli-rich region of the oolemma [62,63]. Genetic perturbation suggests that CD9 is required for microvilli formation [62], which provides an intriguing ultrastructural signature for CD9-associated infertility. These studies exemplify how the resolutions achievable with EM allow it to pinpoint specific, specialized membrane subdomains involved in sperm–egg fusion.

The development of IVF and other ARTs has been accompanied with EM to assess the effects of any treatments on gamete morphology. *In vitro* oocyte maturation is central to these procedures in agricultural species. Comparing oocytes that were *in vitro* maturated to *in vivo* matured by conventional TEM indicated that they are overall similar [64]. Successful cryopreservation of gametes and embryos is crucial for ART and thus its effect was studied extensively [55,65–71]. SEM was used to show that the needle used for intracytoplasmic sperm injection (ICSI) does not damage the ZP or oolemma [72].

## 4. Limitations of conventional EM

Although conventional EM has made invaluable contributions to fertilization research, these methods are limited by the nature of the sample preparation required. Fixation, dehydration, staining and sectioning can all introduce artefacts [17,18], and the resulting micrographs must be interpreted carefully.

Among the most prominent structural distortions that result from conventional EM sample preparation are those associated with aggregation, which can be caused by fixation or by dehydration. Cells are hydrated in their native state, and hydrophilic surfaces of macromolecules are free to interact with water. When water is removed, these surfaces rearrange and may attract each other, forming aggregates [73]. Aggregation explains why empty or clear spaces in the cytoplasm are typical of conventional EM images [17] even though cells are crowded [74].

When using heavy metal stains, the signal we observe comes from the stain deposited on the object and not from the biological material itself. Thus, we can see only as far as the stain penetrates. Apparent differences in density in stained samples are caused by differences in the propensity of different biological materials to be stained, rather than by genuine differences in density. Furthermore, the stain limits achievable resolution to its grain size. Thus, membranes, organelles and large macromolecules are readily observed but most smaller proteins or protein complexes are not.

## 5. Cryo-electron microscopy of cells

The ultimate goal of any imaging method is to capture its subject in a state as close as possible to reality. For cell biology, this translates to imaging cells in a fully hydrated state, free of chemical fixatives or stains. This is precisely the essence of cryo-EM. Cryo-EM is based on rapid vitrification and subsequent imaging of frozen-hydrated specimens at a temperature so low that water does not evaporate significantly in the microscope vacuum.

**Figure 4.**
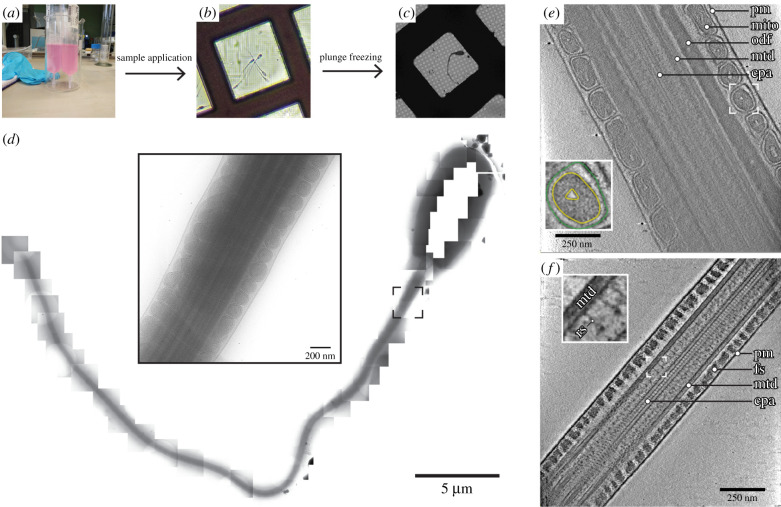
Cryo-ET of pig sperm. (*a*–*c*) The general cryo-ET workflow involves transferring live sperm (*a*) to an EM grid (shown under the light microscope, *b*), followed by plunge-freezing and imaging under a TEM (*c*). (*d*) A montage of cryo-EM projection images tracing a whole pig spermatozoon. A projection image of the midpiece is shown enlarged in the centre. (*e*,*f*) Computational slices through cryo-tomograms of the midpiece (*e*) and the principal piece (*f*). (*e*) In thicker regions of the cell, such as the midpiece, it is possible to resolve fine suborganellar features, such as membranes (inset: green, outer mitochondrial membrane; yellow, inner mitohondrial membrane). (*f*) In thinner regions of the cell, such as the distal part of the principal piece, it is possible to resolve individual protein complexes (inset). In (*e*,*f*), pm, plasma membrane; mito, mitochondrion; odf, outer dense fibre; fs, fibrous sheath; mtd, microtubule doublet; cpa, central pair apparatus; rs, radial spoke.

Cellular samples are prepared for cryo-EM by rapid freezing [75,76]. Cells are cooled so rapidly that they are frozen before water can rearrange to form crystals, thus trapping them in a glass-like (vitreous) state. Small cells can be plunge-frozen simply by dropping them into liquid ethane or ethane/propane [77] cooled to liquid nitrogen temperatures (around −196°C). However, larger cells need to be frozen under high pressure [78] in order to achieve uniform vitrification throughout the cellular volume. For small, free-swimming cells like mammalian sperm, sample preparation for cryo-EM is straightforward and often involves nothing more than pipetting cells onto an EM grid (figure 4*a*–*c*).

Cryo-EM has gained traction in structural biology because of advancements in electron detectors, microscope hardware and image processing [79]. In the so-called single-particle analysis, high-resolution structures of purified proteins can be obtained by averaging many different projection images of individual particles (ideally) randomly oriented in thin, vitreous ice. With parallel advancements in automated data collection and biochemistry, single-particle cryo-EM has become a go-to method for biomolecular structure determination.

Cellular samples pose distinct challenges that necessitate specialized image acquisition and processing strategies. Cells are extremely complex, and the amount of data needed to characterize an entire cell is immense (figure 4*d*). Cells are also extremely variable—no two cells look alike under the EM, which precludes direct averaging procedures. As such, the preferred method for cellular EM is cryo-electron tomography (cryo-ET). In cryo-ET, the sample is tilted in the electron beam and a series of projection images is acquired. Images in the resulting tilt series are then aligned and back-projected to yield a tomogram. Because the sample is tilted in discrete angular steps and to a limited overall angular range, such a tomogram is an incomplete three-dimensional reconstruction of the original object. Despite these limitations, cryo-ET can provide high-resolution three-dimensional information on pleiomorphic objects or rare events, all within the context of a fully hydrated cell. Cryo-ET also provides a uniquely multi-scale capability in that it can resolve membranes and organelles (figure 4*e*) as well as individual protein complexes (figure 4*f*).

As mentioned above, sample thickness is one of the major challenges for TEM. Since most mammalian cells are thicker than a few micrometres, direct imaging by cryo-ET is limited to small cells or to the thin peripheries of larger cells. In recent years, cryo-focused ion beam (FIB) milling [80,81] has emerged as the method-of-choice for fine thinning of cellular material without compression artefacts. Cryo-FIB milling is performed in a dual-beam instrument equipped with both a focused ion beam and an SEM. In these instruments, ions (typically gallium) are accelerated towards the sample, where they sputter material off of the surface, leaving a thin lamella usually approximately 150–250 nm thick. SEM is used for overview imaging, targeting and monitoring the milling process. Lamellae allow high-resolution imaging of the crowded cellular interior [82–86] but represent only a small fraction of the cell's volume [87]. Applying these methods to even larger samples, such as tissues and organisms, is an important challenge driving the next generation of methodological improvements in cryo-sample preparation. Several approaches have yielded promising results [88,89], in some instances allowing for molecular-resolution imaging [90,91].

Unstained biological material consists mainly of elements with low atomic number, which results in very low contrast in the electron microscope. More recently, however, the Volta phase plate (VPP) has emerged as a method of enhancing contrast [92]. The VPP is a heated thin film of amorphous carbon that generates a phase shift of the incident electron beam. The VPP allows high-contrast imaging close to focus, which facilitates the interpretation of cellular tomograms [93].

Because of the sheer complexity of cellular tomograms, they often need to be annotated or segmented in order to aid interpretation. In the past, segmentation was performed by expert annotator manually tracing the structures of interest throughout the tomogram. As data acquisition becomes faster and more reliable, more efficient ways of segmenting tomograms are also necessary. Deep learning and neural network-based approaches have been developed that greatly facilitate the segmentation process [94,95].

When a tomogram contains multiple copies of a given structure, these copies can be aligned and averaged, enhancing signal-to-noise and improving resolution. This process is known as subtomogram averaging, and has the potential to reach subnanometre resolutions in ideal cases [96–98]. Although most applications do not readily reach near-atomic resolutions that are now almost synonymous with single-particle cryo-EM, subtomogram averaging nonetheless yields molecular-resolution maps of proteins or protein complexes that often simply *cannot* be purified. These maps can then be integrated with information from X-ray crystallography, single-particle cryo-EM or structural mass spectrometry to build multi-scale models of large protein complexes within the cellular environment. More importantly, however, cryo-ET combines molecular-resolution information with cellular context, which means that different proteins, protein complexes, or even conformational states or assembly intermediates, can be traced back to their positions in the original tomograms [86,99,100].

## 6. Cryo-electron microscopy is poised to make important contributions to the study of mammalian fertilization

**Figure 5.**
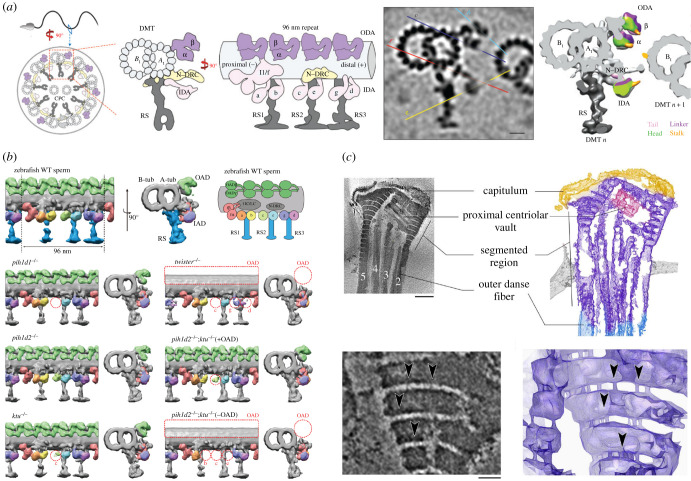
Computational image processing by subtomogram averaging and segmentation aid the analysis and interpretation of cryo-ET data. (*a*) Subtomogram averaging resolved the molecular architecture of doublet microtubules in the axoneme of sea urchin sperm, including positions and conformations of the dynein arms that drive flagellar motility [101]. Scale bar: 10 nm. (*b*) Combined with genetic perturbation, subtomogram averaging defined the roles of individual dynein assembly factors in the organization of the zebrafish sperm axoneme [102]. (*c*) Three-dimensional segmentation unveiled the complex organization of the isolated bovine sperm connecting piece, revealing novel fibrous linkers between plates of the segmented columns [103]. Scale bars: upper left panel, 250 nm; lower left panel, 50 nm.

Cryo-ET and subtomogram averaging have already been applied extensively to the study of sea urchin sperm (figure 5*a*), mostly in the context of the flagellar axoneme [100,101,104,105]. These elegant structural studies defined the molecular architecture of the axoneme, revealing structural motifs that are highly conserved across motile cilia from algae to protists to metazoans [104,106,107]. More recently, advanced image classification methods applied on cryo-tomograms of actively swimming sea urchin sperm unveiled structural rearrangements associated with various stages of the dynein power stroke [101]. Mapping individual dynein states back into their spatial context revealed that most dyneins were in a primed, active conformation and that asymmetric inhibition of dyneins on opposite sides of the flagellum controls bending [100]. These studies exemplify how the true power of cryo-ET lies in the ability to relate molecular structures to precise subcellular contexts.

Cryo-ET also provides a means to assess the molecular effects of targeted genetic disruption. Studies in zebrafish revealed how mutations in different dynein assembly factors resulted in structural defects at specific positions in the axoneme (figure 5*b*), which could be related to altered patterns of sperm motility [102]. Genetic perturbation can also have larger-scale effects on morphology, and the multi-scale capability of cryo-ET allows it to capture these as well. For instance, cryo-scanning transmission electron microscopy (STEM) tomography revealed that *Outer dense fibre 2* (*Odf2*)-haploinsufficient mice were characterized by a unique form of head-midpiece separation [108].

Cryo-ET studies on mammalian sperm are scarce, not least because the accessory structures characteristic of mammalian sperm flagella make them fairly thick compared to other sperm. Nonetheless, where it has been attempted, cryo-ET has already led to discoveries of novel cellular structures, providing a vital resource for future work. For instance, cryo-ET has been applied to thin endpieces of human [109,110] and bovine sperm [110]. These studies revealed the presence of a helical microtubule inner protein (MIP) inside endpiece singlet microtubules [109], and found that doublet-to-singlet transitions can proceed through at least three distinct configurations [110]. There is very little structural information on the distal regions of flagella and cilia in general, probably because they are difficult to capture in thin sections [111], but cryo-ET can circumvent this.

Even for a cell as small as the sperm, thicker regions have remained inaccessible to direct cryo-ET. Structural studies of the connecting piece relied on fractionation [103] to generate samples thin enough for high-resolution imaging. This work used three-dimensional segmentation to reveal the complexity of the large, asymmetric connecting piece. Cryo-ET revealed fine linkers between individual plates of the segmented columns that could be involved in elastic deformation of the structure during sperm movement (figure 5*c*).

**Figure 6.**
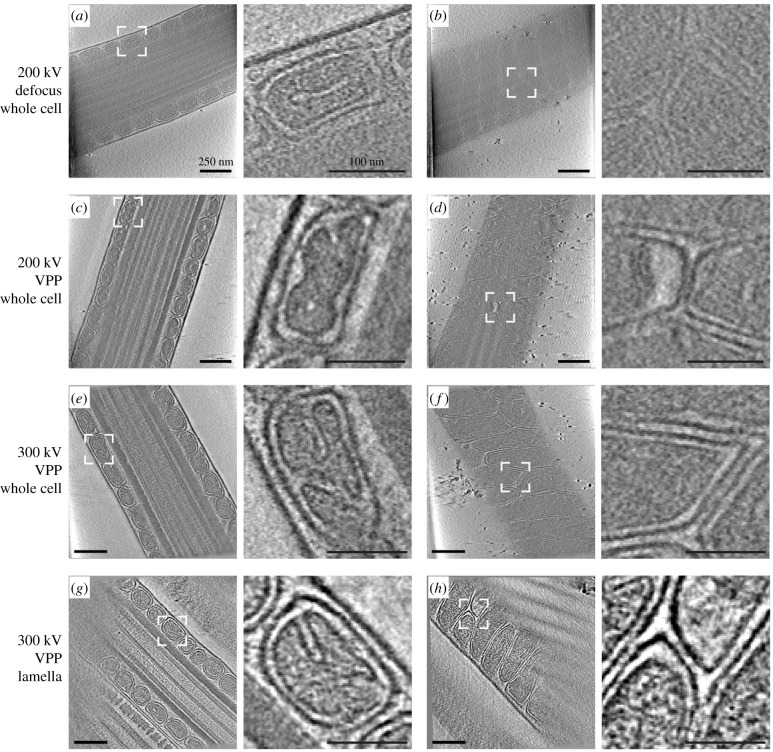
Cryo-FIB milling and use of the VPP improve cryo-ET imaging. Slices through cryo-tomograms of pig sperm midpieces, acquired (*a*,*b*) on whole cells with an accelerating voltage of 200 kV and without the VPP; (*c*,*d*) on whole cells with an accelerating voltage of 200 kV and with the VPP; (*e*,*f*) on whole cells with an accelerating voltage of 300 kV and with the VPP; (*g*,*h*) on lamellae (approx. 300 nm thick) with an accelerating voltage of 300 kV and with the VPP. Left panels (*a*,*c*,*e*,*g*) show central longitudinal slices, while right panels (*b*,*d*,*f*,*h*) show tangential slices. Scale bars: left panels, 250 nm; right panels (digital zooms), 100 nm.

We here show how the recent aforementioned developments in cryo-ET sample preparation, imaging and processing now enable studies of whole, intact mammalian sperm (figure 6). For instance, VPP imaging of whole pig sperm reveals the three-dimensional organization of the mitochondrial sheath (figure 6*c*–*f*). Imaging cryo-FIB-milled pig sperm further reveals the internal organization of mitochondrial cristae (figure 6*g*,*h*). Individual protein complexes, such as the axonemal radial spokes, can be easily identified in VPP tomograms of slightly thinner regions like the principal piece (figure 4*f*).

Beyond detailed characterization of sperm structure, cryo-EM also has the potential to interrogate sperm function. An intriguing example of this is how cryo-EM was used to study post-ejaculatory modifications associated with mosquito sperm, revealing drastic changes in the organization of the glycocalyx that correlate with changes in sperm motility and female fertility [112]. Because rapid vitrification provides superior sample preservation to conventional EM preparations, cryo-EM/ET would be particularly useful for re-investigating the membrane remodelling events associated with capacitation and the acrosome reaction in mammalian sperm. Cryo-EM/ET could also be used to interrogate surface changes that occur during the formation and dissociation of sperm aggregates, such as in certain monotreme [113] or rodent [114] species.

Cryo-EM is beginning to make inroads into studying oocyte biology, as well. Single-particle cryo-EM of purified tetraspanin CD9 in complex with a partner protein provided potential mechanisms for CD9-mediated assembly of cell surface microdomains [115]. Helical reconstruction of purified uromodulin filaments revealed a structural mechanism for the assembly of ZP-domain containing proteins [116], setting the stage for defining how the egg coat assembles and interacts with sperm.

## 7. Challenges and emerging solutions in electron microscopy of gametes

### 7.1. Electron microscopy of large volumes

Although cryo-FIB milling makes thicker cells accessible to cryo-ET, it is still limited to samples that can be reliably vitrified by plunge-freezing (that is, a few micrometres thick). This precludes the analysis of larger cells, like the oocyte, and tissues, like the testes. Several groups are actively developing methods to address these limitations. One promising strategy involves first producing approximately 20 µm thick slabs from high-pressure-frozen samples either with a cryo-ultramicrotome or a cryo-FIB/SEM, followed by finer milling with a cryo-FIB/SEM to 150–300 nm [90,91]. Currently, however, these methods are technically challenging and low-throughput. Furthermore, even when milling and transfer steps are successful, each 150 nm thick lamella represents a volume of only approximately 0.24 µm^3^ [87], which is only approximately 0.00005% of the volume of a 100 µm oocyte. As such, there will be biological questions for which cryo-FIB-ET simply will not be the method of choice.

A family of EM methods known as volume EM would be applicable in cases where entire cells or tissues need to be imaged. Volume EM encompasses methods such as serial block-face electron microscopy (SBEM), focused ion beam scanning electron microscopy (FIB/SEM), and automated tape-collecting ultramicrotome scanning electron microscopy (ATUM-SEM) [117]. In SBEM, a microtome is mounted inside the microscope chamber and a diamond knife is used to shave a thin layer off the top of the sample, which is then imaged by SEM. In FIB/SEM, a gallium beam is used instead of a diamond knife, allowing for finer milling on the order of 2–5 nm. In ATUM-SEM, the entire sample is first sectioned outside the microscope with a diamond knife, and the sections are automatically collected on tape, which can then be loaded into an SEM. These volume EM methods have been applied to muscle biology [118,119], large-scale connectomics [120–122] and recently to oocyte biology as well [123,124].

Although most applications of volume EM still use conventional EM sample preparation, these methods have also recently started to interface with cryo-EM. FIB/SEM in particular can be used to image high-pressure-frozen samples at cryo-temperatures [125]. Among other applications, cryo-FIB/SEM has been used for large-scale comparative studies of biomineralizing protists [126], and for whole-cell ultrastructural studies directly on patient samples [127].

### 7.2. Correlative light and electron microscopy

Because contrast in cryo-EM comes directly from biological material, EM data capture everything, not just the signal from a protein of interest. Some large protein complexes, such as ATP synthase and the nuclear pore complex, can be recognized directly by virtue of their distinct shapes and by the fact that they occupy defined subcellular locations [84,128]. However, it is often difficult to identify individual densities in information-rich cellular tomograms.

One solution to the localization problem is to integrate the molecular specificity of fluorescence microscopy into the EM pipeline. This technique is called correlative light and electron microscopy (CLEM) and has in fact been applied quite successfully for conventional TEM-based methods [4,129]. However, cryo-CLEM is much more difficult. The technical challenges involved in optimizing the optical set-up while minimizing chances for ice contamination and sample devitrification cannot be overstated. Several promising approaches now exist, including light microscopes integrated within the FIB/SEM chamber [130] and algorithms that enable super-resolution CLEM with low laser intensities [131]. Cryo-FIB/SEM-based ‘mill-and-view’ strategies can also be integrated with cryo-fluorescence microscopy to define sites of interest for subsequent cryo-FIB milling and high-resolution cryo-ET [132]. Volume EM has also been interfaced with super-resolution microscopy, enabling quantitative studies of ultrastructure across entire cells [133].

Because CLEM methods often involve genetic tags and thus require the system to be genetically manipulable, they will always be challenging to apply to gamete biology. Although mRNA microinjection is a viable alternative for oocytes, sperm pose a more difficult challenge as they are transcriptionally and translationally silent. Surface proteins can be targeted through antibody- or nanobody-based labelling, or through aptamer-based DNA origami strategies. However, most applications will probably involve the generation of transgenic animal lines, and thus will benefit greatly from efforts to extend the range of species for which genetic tools are available [134].

## 8. Concluding remarks

Since the first electron microscope image of eukaryotic cells taken in 1945 [135], EM has enabled biologists to peer deeper into the cell than ever before. Our modern understanding of cell biology is built on foundations laid by EM-based structural work [136–138]. Gamete biology is no exception—EM extended our understanding of gamete structure and revealed that structural specialization extends beyond that of overall form, down to the level of individual subcellular components.

From a practical perspective, insights from conventional EM ultimately led to successful IVF and ARTs [14]. However, the harsh sample preparation methods used in conventional EM—fixation, dehydration and staining—can introduce artefacts that distort our vision of cell ultrastructure [17]. Because many processes central to mammalian fertilization, such as capacitation and gamete fusion itself, involve complex rearrangements of membranes and individual protein complexes, conventional EM can only go so far.

Much remains to be learned about gamete structure and how it relates to the biology of fertilization. New and emerging methods in cellular EM are poised to seamlessly bridge the molecular and cellular scales. Examples of unresolved questions in gamete biology that are now ripe for exploration with new EM techniques include: how does membrane remodelling during capacitation and the acrosome reaction transform the mammalian sperm into a fusion-competent cell? How do differences in sperm motility patterns across species arise from variations at the subcellular level? How is the ZP organized in three dimensions and how is it remodelled during oocyte maturation and post-fertilization? Answering some of these questions undoubtedly will not only facilitate developing novel infertility treatments and contraceptives, but also improve ART for biodiversity conservation [139].

## Data accessibility

This article has no additional data.

## References

[1] van Leeuwenhoek A 1677 Observationes D. Anthonii Lewenhoeck, de natis'e semine genitali animalculis. Phil. Trans. R. Soc. 12, 1040–1046. (10.1098/rstl.1677.0068)

[2] von Baer KE. 1827 De ovi mammalium et hominis genesi (On the genesis of the ovum of mammals and of man). Leipzig, Germany: Leopold Voss.

[3] Hertwig O 1876 Beiträge zur kenntniss der bildung, befruchtung und theilung des thierischen eies. Morph. Jahrb. 1, 347–434.

[4] Fishman EL et al 2018 A novel atypical sperm centriole is functional during human fertilization. Nat. Commun. 9, 2210 (10.1038/s41467-018-04678-8) 29880810 PMC5992222

[5] Le Guennec M et al. 2020 A helical inner scaffold provides a structural basis for centriole cohesion. Sci. Adv. 6, eaaz4137 (10.1126/sciadv.aaz4137) 32110738 PMC7021493

[6] Chung J-J, Shim S-H, Everley RA, Gygi SP, Zhuang X, Clapham DE 2014 Structurally distinct Ca^2+^ signaling domains of sperm flagella orchestrate tyrosine phosphorylation and motility. Cell 157, 808–822. (10.1016/j.cell.2014.02.056) 24813608 PMC4032590

[7] Knott G, Genoud C 2013 Is EM dead? J. Cell Sci. 126, 4545–4552. (10.1242/jcs.124123) 24124192

[8] Griffiths G 2004 Ultrastructure in cell biology: do we still need it? Eur. J. Cell Biol. 83, 245–251. (10.1078/0171-9335-00375) 15511081

[9] Marton L 1976 Early application of electron microscopy to biology. Ultramicroscopy 1, 281–296. (10.1016/0304-3991(76)90046-2) 800686

[10] Seymour FI, Benmosche M 1941 Magnification of spermatozoa by means of the electron microscope. J. Am. Med. Assoc. 116, 2489 (10.1001/jama.1941.62820220001008)

[11] Reed CI, Reed BP 1948 Comparative study of human and bovine sperm by electron microscopy. Anat. Rec. 100, 1–7. (10.1002/ar.1091000102) 18917637

[12] Fawcett DW 1970 A comparative view of sperm ultrastructure. Biol. Reprod. Suppl. 2, 90–127. 12254595

[13] Fawcett DW 1975 The mammalian spermatozoon. Dev. Biol. 44, 394–436. 805734 10.1016/0012-1606(75)90411-x

[14] Schwartz P, Hinney B, Nayudu PL, Michelmann HW 2003 Oocyte-sperm interaction in the course of IVF: a scanning electron microscopy analysis. Reprod. Biomed. Online 7, 205–210. (10.1016/S1472-6483(10)61753-1) 14567893

[15] Camboni A, Martinez-Madrid B, Dolmans MM, Amorim CA, Nottola SA, Donnez J, Van Langendonckt A. 2008 Preservation of fertility in young cancer patients: contribution of transmission electron microscopy. Reprod. Biomed. Online 17, 136–150. (10.1016/S1472-6483(10)60303-3) 18616902

[16] Harris JR 2015 Transmission electron microscopy in molecular structural biology: a historical survey. Arch. Biochem. Biophys. 581, 3–18. (10.1016/j.abb.2014.11.011) 25475529

[17] Dubochet J, Zuber B, Eltsov M, Bouchet-Marquis C, Al-Amoudi A, Livolant F 2007 How to ‘read’ a vitreous section. Methods Cell Biol. 79, 385–406. (10.1016/S0091-679X(06)79015-X) 17327166

[18] Pilhofer M, Ladinsky MS, McDowall AW, Jensen GJ 2010 Bacterial TEM: new insights from cryo-microscopy. Amsterdam, The Netherlands: Elsevier Inc. 10.1016/S0091-679X(10)96002-0 20869517

[19] Schatten H 2010 The role of scanning electron microscopy in cell and molecular biology: SEM basics, past accomplishments, and new frontiers. In Scanning electron microscopy for the life sciences (ed. Schatten H), pp. 1–15. Cambridge, UK: Cambridge University Press.

[20] Grange M, Vasishtan D, Grünewald K 2017 Cellular electron cryo tomography and in situ sub-volume averaging reveal the context of microtubule-based processes. J. Struct. Biol. 197, 181–190. (10.1016/j.jsb.2016.06.024) 27374320 PMC5287354

[21] Winey M, Meehl JB, O'Toole ET, Giddings TH 2014 Conventional transmission electron microscopy. Mol. Biol. Cell 25, 319–323. (10.1091/mbc.E12-12-0863) 24482357 PMC3907272

[22] Tokuyasu KT 1973 A technique for ultracryotomy of cell suspensions and tissues. J. Cell Biol. 57, 551–565. (10.1083/jcb.57.2.551) 4121290 PMC2108989

[23] Steinbrecht RA, Müller M 1987 Freeze-substitution and freeze-drying. In Cryotechniques in biological electron microscopy (eds Steinbrecht RA, Zierold K), pp. 149–172. Berlin, Germany: Springer.

[24] Harvey DMR 1982 Freeze-substitution. J. Microsc. 127, 209–221. (10.1111/j.1365-2818.1982.tb00414.x) 6750131

[25] Heuser JE 2011 The origins and evolution of freeze-etch electron microscopy. J. Electron Microsc. (Tokyo) 60, 3–29. (10.1093/jmicro/dfr044) PMC3202940 21844598

[26] Hall CE 1956 Visualization of individual macromolecules with the electron microscope. Proc. Natl Acad. Sci. USA 42, 801–806. (10.1073/pnas.42.11.801)

[27] Mortimer D 1994 Practical laboratory andrology. New York, NY: Oxford University Press.

[28] Hafez ESE, Kanagawa H 1973 Scanning electron microscopy of human, monkey and rabbit spermatozoa. Fertil. Steril. 24, 776–787. (10.1016/S0015-0282(16)39973-3) 4200433

[29] Koehler JK 1972 Human sperm head ultrastructure: a freeze-etching study. J. Ultrasruct. Res. 39, 520–539. (10.1016/S0022-5320(72)90118-9) 4556325

[30] Albertini DF 2015 The mammalian oocyte. In Knobil and Neill's physiology of reproduction (eds Plant TM, Zeleznik AJ), pp. 59–97, 4th edn San Diego, CA: Academic Press.

[31] Familiari G 2008 Structural changes of the zona pellucida during fertilization and embryo development. Front. Biosci. 13, 6730 (10.2741/3185) 18508691

[32] Familiari G, Relucenti M, Heyn R, Micara G, Correr S 2006 Three-dimensional structure of the zona pellucida at ovulation. Microsc. Res. Tech. 69, 415–426. (10.1002/jemt.20301) 16703610

[33] Magerkurth C 1999 Scanning electron microscopy analysis of the human zona pellucida: influence of maturity and fertilization on morphology and sperm binding pattern. Hum. Reprod. 14, 1057–1066. (10.1093/humrep/14.4.1057) 10221241

[34] Sathananthan AH, Trounson AO, Wood C, Leeton JF 1982 Ultrastructural observations on the penetration of human sperm into the zona pellucida of the human egg in vitro. J. Androl. 3, 356–364. (10.1002/j.1939-4640.1982.tb00702.x)

[35] Nicosia SV, Wolf DP, Inoue M 1977 Cortical granule distribution and cell surface characteristics in mouse eggs. Dev. Biol. 57, 56–74. (10.1016/0012-1606(77)90354-2) 863112

[36] Primakoff P, Myles DG 2007 Cell-cell membrane fusion during mammalian fertilization. FEBS Lett. 581, 2174–2180. (10.1016/j.febslet.2007.02.021) 17328899

[37] Pleuger C, Lehti MS, Dunleavy JE, Fietz D, O'Bryan MK 2020 Haploid male germ cells—the Grand Central Station of protein transport. Hum. Reprod. Update 26, 474–500. (10.1093/humupd/dmaa004) 32318721

[38] Mortimer D 2018 The functional anatomy of the human spermatozoon: relating ultrastructure and function. Mol. Hum. Reprod. 24, 567–592. (10.1093/molehr/gay040) 30215807

[39] Phillips DM 1977 Mitochondrial disposition in mammalian spermatozoa. J. Ultrasruct. Res. 58, 144–154. (10.1016/S0022-5320(77)90026-0) 402480

[40] Bahr GF, Engler WF 1970 Considerations of volume, mass, DNA, and arrangement of mitochondria in the midpiece of bull spermatozoa. Exp. Cell Res. 60, 338–340. (10.1016/0014-4827(70)90526-4) 5422964

[41] Chen Y et al 2017 Glycerol kinase-like proteins cooperate with Pld6 in regulating sperm mitochondrial sheath formation and male fertility. Cell Discov. 3, 1–8. (10.1038/celldisc.2017.30) PMC5566117 28852571

[42] Bartoov B, Eltes F, Weissenberg R, Lunenfeld B 1980 Morphological characterization of abnormal human spermatozoa using transmission electron microscopy. Arch. Androl. 5, 305–322. (10.3109/01485018008987000) 7447532

[43] Chemes HE 2017 Sperm ultrastructure in fertile men and male sterility: revisiting teratozoospermia. In The sperm cell: production, maturation, fertilization, regeneration (eds De Jonge CJ, Barratt CLR), pp. 36–58. Cambridge, UK: Cambridge University Press.

[44] Gopalkrishnan K, Anand Kumar TC 1990 Scanning electron microscopy in the assessment of sperm morphology. Indian J. Med. Res. 92, 169–174. 2401537

[45] Grab D, Thierauf S, Rosenbusch B, Sterzik K 1993 Scanning electron microscopy of human sperms after preparation of semen for in-vitro fertilization. Arch. Gynecol. Obstet. 252, 137–141. (10.1007/BF02456677) 8503704

[46] Liakatas J, Williams AE, Hargreave TB 1982 Scoring sperm morphology using the scanning electron microscope. Fertil. Steril. 38, 227–232. (10.1016/S0015-0282(16)46464-2) 7106317

[47] Gu NH, Zhao WL, Wang GS, Sun F 2019 Comparative analysis of mammalian sperm ultrastructure reveals relationships between sperm morphology, mitochondrial functions and motility. Reprod. Biol. Endocrinol. 17, 1–12. (10.1186/s12958-019-0510-y) 31416446 PMC6696699

[48] Yanagimachi R 1981 Mechanisms of fertilization in mammals. In Fertilization and embryonic development in vitro (eds Mastroianni L, Biggers JD), pp. 81–182. Boston, MA: Springer.

[49] Nagae T, Yanagimachi R, Srivastava PN, Yanagimachi H 1986 Acrosome reaction in human spermatozoa. Fertil. Steril. 45, 701–707. (10.1016/S0015-0282(16)49344-1) 3699172

[50] Russell L, Peterson R, Freund M 1979 Direct evidence for formation of hybrid vesicles by fusion of plasma and outer acrosomal membranes during the acrosome reaction in boar spermatozoa. J. Exp. Zool. 208, 41–55. (10.1002/jez.1402080106) 381567

[51] Aguas AP, Pinto Da Silva P 1989 Bimodal redistribution of surface transmembrane glycoproteins during Ca^2+^-dependent secretion (acrosome reaction) in boar spermatozoa. J. Cell Sci. 93, 467–479. 2606938 10.1242/jcs.93.3.467

[52] Baca M, Zamboni L 1967 The fine structure of human follicular oocytes. J. Ultrastruct. Res. 19, 354–381. (10.1016/S0022-5320(67)80225-9) 6053018

[53] Paulini F, Silva RC, De Paula Rôlo JLJ, Lucci CM. 2014 Ultrastructural changes in oocytes during folliculogenesis in domestic mammals. J. Ovarian Res. 7, 1–12. (10.1186/s13048-014-0102-6) 25358389 PMC4224757

[54] Dadarwal D, Adams GP, Hyttel P, Brogliatti GM, Caldwell S, Singh J 2015 Organelle reorganization in bovine oocytes during dominant follicle growth and regression. Reprod. Biol. Endocrinol. 13, 1–11. (10.1186/s12958-015-0122-0) 26577904 PMC4650271

[55] Diez C et al 2005 Bovine oocyte vitrification before or after meiotic arrest: effects on ultrastructure and developmental ability. Theriogenology 64, 317–333. (10.1016/j.theriogenology.2004.11.023) 15955356

[56] Wassarman PM, Litscher ES 2016 A bespoke coat for eggs: getting ready for fertilization. In Essays on development biology, part B: PMBT-CT (ed. Wassarman DB), pp. 539–552. San Diego, CA: Academic Press. 10.1016/bs.ctdb.2015.10.018 26969999

[57] Wassarman PM 2008 Zona pellucida glycoproteins. J. Biol. Chem. 283, 24 285–24 289. (10.1074/jbc.R800027200) PMC2528931 18539589

[58] Greve JM, Wassarman PM 1985 Mouse egg extracellular coat is a matrix of interconnected filaments possessing a structural repeat. J. Mol. Biol. 181, 253–264. (10.1016/0022-2836(85)90089-0) 3845123

[59] Funahashi H, Ekwall H, Kikuchi K, Rodriguez-Martinez H 2001 Transmission electron microscopy studies of the zona reaction in pig oocytes fertilized in vivo and in vitro. Reproduction 122, 443–452. (10.1530/rep.0.1220443) 11597309

[60] Talbot P, Dicarlantonio G 1984 The oocyte-cumulus complex: ultrastructure of the extracellular components in hamsters and mice. Gamete Res. 10, 127–142. (10.1002/mrd.1120100205)

[61] Yanagimachi R, Noda YD 1970 Electron microscope studies of sperm incorporation into the golden hamster egg. Am. J. Anat. 128, 429–461. (10.1002/aja.1001280404) 4194878

[62] Runge KE, Evans JE, He ZY, Gupta S, McDonald KL, Stahlberg H, Primakoff P, Myles DG 2007 Oocyte CD9 is enriched on the microvillar membrane and required for normal microvillar shape and distribution. Dev. Biol. 304, 317–325. (10.1016/j.ydbio.2006.12.041) 17239847

[63] Bianchi E, Doe B, Goulding D, Wright GJ 2014 Juno is the egg Izumo receptor and is essential for mammalian fertilization. Nature 508, 483–487. (10.1038/nature13203) 24739963 PMC3998876

[64] Segovia Y, Victory N, Peinado I, García-Valverde LM, García M, Aizpurua J, Monzó A, Gómez-Torres MJ 2017 Ultrastructural characteristics of human oocytes vitrified before and after in vitro maturation. J. Reprod. Dev. 63, 377–382. (10.1262/jrd.2017-009) 28458301 PMC5593089

[65] Shahedi A, Hosseini A, Khalili MA, Norouzian M, Salehi M, Piriaei A, Nottola SA 2013 The effect of vitrification on ultrastructure of human in vitro matured germinal vesicle oocytes. Eur. J. Obstet. Gynecol. Reprod. Biol. 167, 69–75. (10.1016/j.ejogrb.2012.11.006) 23260597

[66] Khalili MA, Maione M, Palmerini MG, Bianchi S, Macchiarelli G, Nottola SA 2012 Ultrastructure of human mature oocytes after vitrification. Eur. J. Histochem. 56, 38 (10.4081/ejh.2012.e38) PMC3493984 23027354

[67] Palmerini MG, Antinori M, Maione M, Cerusico F, Versaci C, Nottola SA, Macchiarelli G, Khalili MA, Antinori S 2014 Ultrastructure of immature and mature human oocytes after cryotop vitrification. J. Reprod. Dev. 60, 411–420. (10.1262/jrd.2014-027) 25168087 PMC4284314

[68] Leonel ECR et al 2019 Stepped vitrification technique for human ovarian tissue cryopreservation. Sci. Rep. 9, 1–12. (10.1038/s41598-019-56585-7) 31882972 PMC6934833

[69] Gosden RG, Yin H, Bodine RJ, Morris GJ 2010 Character, distribution and biological implications of ice crystallization in cryopreserved rabbit ovarian tissue revealed by cryo-scanning electron microscopy. Hum. Reprod. 25, 470–478. (10.1093/humrep/dep395) 19933523

[70] Ozkavukcu S, Erdemli E, Isik A, Oztuna D, Karahuseyinoglu S 2008 Effects of cryopreservation on sperm parameters and ultrastructural morphology of human spermatozoa. J. Assist. Reprod. Genet. 25, 403–411. (10.1007/s10815-008-9232-3) 18704674 PMC2582121

[71] Agha-Rahimi A, Khalili MA, Nottola SA, Miglietta S, Moradi A 2016 Cryoprotectant-free vitrification of human spermatozoa in new artificial seminal fluid. Andrology 4, 1037–1044. (10.1111/andr.12212) 27566065

[72] Schwartz P, Magerkurth C, Michelmann HW 1996 Scanning electron microscopy of the zona pellucida of human oocytes during intracytoplasmic sperm injection (ICSI). Hum. Reprod. 11, 2693–2696. (10.1093/oxfordjournals.humrep.a019193) 9021374

[73] Kellenberger E, Kistler J 1979 The physics of specimen preparation. In Unconventional electron microscopy for molecular structure determination (eds Hoppe W, Mason R), pp. 49–79. Wiesbaden, Germany: Vieweg + Teubner Verlag.

[74] Goodsell DS 2009 The machinery of life. New York, NY: Springer.

[75] Dubochet J, Lepault J, Freeman R, Berriman JA, Homo J-C 1982 Electron microscopy of frozen water and aqueous solutions. J. Microsc. 128, 219–237. (10.1111/j.1365-2818.1982.tb04625.x)

[76] Lepault J, Dubochet J 1986 Electron microscopy of frozen hydrated specimens: preparation and characteristics. Methods Enzymol. 127, 719–730. (10.1016/0889-1605(86)90041-8) 3736433

[77] Tivol WF, Briegel A, Jensen GJ 2008 An improved cryogen for plunge freezing. Microsc. Microanal. 14, 375–379. (10.1017/S1431927608080781) 18793481 PMC3058946

[78] Dubochet J 1995 High-pressure freezing for cryoelectron microscopy. Trends Cell Biol. 5, 366–368. (10.1016/S0962-8924(00)89071-6) 14732080

[79] Glaeser RM 2019 How good can single-particle cryo-EM become? What remains before it approaches its physical limits? Annu. Rev. Biophys. 48, 45–61. (10.1146/annurev-biophys-070317-032828) 30786229

[80] Marko M, Hsieh C, Schalek R, Frank J, Mannella C 2007 Focused-ion-beam thinning of frozen-hydrated biological specimens for cryo-electron microscopy. Nat. Methods 4, 215–217. (10.1038/nmeth1014) 17277781

[81] Rigort A, Bäuerlein FJB, Villa E, Eibauer M, Laugks T, Baumeister W, Plitzko JM 2012 Focused ion beam micromachining of eukaryotic cells for cryoelectron tomography. Proc. Natl Acad. Sci. USA 109, 4449–4454. (10.1073/pnas.1201333109) 22392984 PMC3311327

[82] Engel BD, Schaffer M, Cuellar LK, Villa E, Plitzko JM, Baumeister W 2015 Native architecture of the chlamydomonas chloroplast revealed by *in situ* cryo-electron tomography. eLife 2015, 1–29. (10.7554/eLife.04889) PMC4568591 26367339

[83] Engel BD, Schaffer M, Albert S, Asano S, Plitzko JM, Baumeister W 2015 In situ structural analysis of Golgi intracisternal protein arrays. Proc. Natl Acad. Sci. USA 112, 11 264–11 269. (10.1073/pnas.1515337112) PMC4568700 26311849

[84] Mahamid J et al 2016 Visualizing the molecular sociology at the HeLa cell nuclear periphery. Science 351, 969–972. (10.1126/science.aad8857) 26917770

[85] Weiss GL, Kieninger A-K, Maldener I, Forchhammer K, Pilhofer M 2019 Structure and function of a bacterial gap junction analog. Cell 178, 374–384. (10.1101/462465) 31299201 PMC6630896

[86] Asano S, Fukuda Y, Beck F, Aufderheide A, Förster F, Danev R, Baumeister W 2015 A molecular census of 26S proteasomes in intact neurons. Science 347, 439–442. (10.1126/science.1261197) 25613890

[87] Rast A, Schaffer M, Albert S, Wan W, Pfeffer S, Beck F, Plitzko JM, Nickelsen J, Engel BD 2019 Biogenic regions of cyanobacterial thylakoids form contact sites with the plasma membrane. Nat. Plants 5, 436–446. (10.1038/s41477-019-0399-7) 30962530

[88] Harapin J, Börmel M, Sapra KT, Brunner D, Kaech A, Medalia O 2015 Structural analysis of multicellular organisms with cryo-electron tomography. Nat. Methods 12, 634–636. (10.1038/nmeth.3401) 25961413

[89] Mahamid J, Schampers R, Persoon H, Hyman AA, Baumeister W, Plitzko JM 2015 A focused ion beam milling and lift-out approach for site-specific preparation of frozen-hydrated lamellas from multicellular organisms. J. Struct. Biol. 192, 262–269. (10.1016/j.jsb.2015.07.012) 26216184

[90] Schaffer M et al 2019 A cryo-FIB lift-out technique enables molecular-resolution cryo-ET within native *Caenorhabditis elegans* tissue. Nat. Methods 16, 757–762. (10.1038/s41592-019-0497-5) 31363205

[91] Zhang J, Zhang D, Sun L, Ji G, Huang X, Niu T, Sun F 2019 VHUT-cryo-FIB, a method to fabricate frozen-hydrated lamella of tissue specimen for in situ cryo-electron tomography. *bioRxiv* 727149 (10.1101/727149)

[92] Danev R, Buijsse B, Khoshouei M, Plitzko JM, Baumeister W 2014 Volta potential phase plate for in-focus phase contrast transmission electron microscopy. Proc. Natl Acad. Sci. USA 111, 15 635–15 640. (10.1073/pnas.1418377111) 25331897 PMC4226124

[93] Fukuda Y, Laugks U, Lučić V, Baumeister W, Danev R 2015 Electron cryotomography of vitrified cells with a Volta phase plate. J. Struct. Biol. 190, 143–154. (10.1016/j.jsb.2015.03.004) 25770733

[94] Chen M, Dai W, Sun SY, Jonasch D, He CY, Schmid MF, Chiu W, Ludtke SJ 2017 Convolutional neural networks for automated annotation of cellular cryo-electron tomograms. Nat. Methods 14, 983–985. (10.1038/nmeth.4405) 28846087 PMC5623144

[95] Zeng X, Leung MR, Zeev-Ben-Mordehai T, Xu M 2018 A convolutional autoencoder approach for mining features in cellular electron cryo-tomograms and weakly supervised coarse segmentation. J. Struct. Biol. 202, 150–160. (10.1016/j.jsb.2017.12.015) 29289599 PMC6661905

[96] Tegunov D, Xue L, Dienemann C, Cramer P, Mahamid J. 2020 Multi-particle cryo-EM refinement with M visualizes ribosome-antibiotic complex at 3.7-Å inside cells. *bioRxiv*. 10.1038/s41592-020-01054-7 PMC7611018 33542511

[97] Sutton G, Sun D, Fu X, Kotecha A, Hecksel CW 2020 Assembly intermediates of orthoreovirus captured in the cell. *bioRxiv*.

[98] O'Reilly FJ et al 2020 In-cell architecture of an actively transcribing-translating expressome. Science 369, 554–557. (10.1126/science.abb3758) 32732422 PMC7115962

[99] Albert S et al 2020 Direct visualization of degradation microcompartments at the ER membrane. Proc. Natl Acad. Sci. USA 117, 1069–1080. (10.1073/pnas.1905641117) 31882451 PMC6969544

[100] Lin J, Nicastro D 2018 Asymmetric distribution and spatial switching of dynein activity generates ciliary motility. Science 360, eaar1968 (10.1126/science.aar1968) 29700238 PMC6640125

[101] Lin J, Okada K, Raytchev M, Smith MC, Nicastro D 2014 Structural mechanism of the dynein power stroke. Nat. Cell Biol. 16, 479–485. (10.1038/ncb2939) 24727830 PMC4102432

[102] Yamaguchi H, Oda T, Kikkawa M, Takeda H 2018 Systematic studies of all PIH proteins in zebrafish reveal their distinct roles in axonemal dynein assembly. eLife 7, 1–25. (10.7554/elife.36979) PMC6008050 29741156

[103] Ounjai P, Kim KD, Lishko PV, Downing KH 2012 Three-dimensional structure of the bovine sperm connecting piece revealed by electron cryotomography. Biol. Reprod. 87, 73 (10.1095/biolreprod.112.101980) 22767409 PMC3464910

[104] Nicastro D, Fu X, Heuser T, Tso A, Porter ME, Linck RW 2011 Cryo-electron tomography reveals conserved features of doublet microtubules in flagella. Proc. Natl Acad. Sci. USA 108, E845–E853. (10.1073/pnas.1106178108) 21930914 PMC3198354

[105] Carbajal-González BI, Heuser T, Fu X, Lin J, Smith BW, Mitchell DR, Nicastro D 2013 Conserved structural motifs in the central pair complex of eukaryotic flagella. Cytoskeleton 70, 101–120. (10.1002/cm.21094) 23281266 PMC3914236

[106] Nicastro D, Schwartz C, Pierson J, Gaudette R, Porter ME, McIntosh JR 2006 The molecular architecture of axonemes revealed by cryoelectron tomography. Science 313, 944–948. (10.1126/science.1128618) 16917055

[107] Pigino G, Maheshwari A, Bui KH, Shingyoji C, Kamimura S, Ishikawa T 2012 Comparative structural analysis of eukaryotic flagella and cilia from *Chlamydomonas*, *Tetrahymena*, and sea urchins. J. Struct. Biol. 178, 199–206. (10.1016/j.jsb.2012.02.012) 22406282

[108] Ito C et al 2019 Odf2 haploinsufficiency causes a new type of decapitated and decaudated spermatozoa, Odf2-DDS, in mice. Sci. Rep. 9, 1–13. (10.1038/s41598-019-50516-2) 31582806 PMC6776547

[109] Zabeo D, Heumann JM, Schwartz CL, Suzuki-Shinjo A, Morgan G, Widlund PO, Höög JL 2018 A lumenal interrupted helix in human sperm tail microtubules. Sci. Rep. 8, 1–11. (10.1038/s41598-018-21165-8) 29426884 PMC5807425

[110] Zabeo D, Croft JT, Höög JL 2019 Axonemal doublet microtubules can split into two complete singlets in human sperm flagellum tips. FEBS Lett. 593, 892–902. (10.1002/1873-3468.13379) 30959570 PMC6594080

[111] Fisch C, Dupuis-Williams P 2011 Ultrastructure of cilia and flagella—back to the future! Biol. Cell 103, 249–270. (10.1042/BC20100139) 21728999

[112] Noble JM, Degner EC, Harrington LC, Kourkoutis LF 2019 Cryo-electron microscopy reveals that sperm modification coincides with female fertility in the mosquito *Aedes aegypti*. Sci. Rep. 9, 1–11. (10.1038/s41598-019-54920-6) 31811199 PMC6898104

[113] Nixon B, Ecroyd H, Dacheux J-L, Dacheux F, Labas V, Johnston SD, Jones RC 2016 Formation and dissociation of sperm bundles in monotremes. Biol. Reprod. 95, 91 (10.1095/biolreprod.116.140491) 27557648

[114] Fisher HS, Giomi L, Hoekstra HE, Mahadevan L 2014 The dynamics of sperm cooperation in a competitive environment. Proc. R. Soc. Lond. B 281, 20140296 (10.1098/rspb.2014.0296) PMC4123693 25056618

[115] Umeda R et al 2020 Structural insights into tetraspanin CD9 function. Nat. Commun. 11, 1–7. (10.1038/s41467-020-15459-7) 32231207 PMC7105497

[116] Stsiapanava A et al 2020 Cryo-EM structure of native human uromodulin, a zona pellucida module polymer. *bioRxiv* (10.1101/2020.05.28.119206)

[117] Titze B, Genoud C 2016 Volume scanning electron microscopy for imaging biological ultrastructure. Biol. Cell 108, 307–323. (10.1111/boc.201600024) 27432264

[118] Glancy B, Hartnell LM, Malide D, Yu ZX, Combs CA, Connelly PS, Subramaniam S, Balaban RS 2015 Mitochondrial reticulum for cellular energy distribution in muscle. Nature 523, 617–620. (10.1038/nature14614) 26223627 PMC6988728

[119] Vincent AE et al 2019 Quantitative 3D mapping of the human skeletal muscle mitochondrial network. Cell Rep. 26, 996–1009.e4. (10.1016/j.celrep.2019.01.010) 30655224 PMC6513570

[120] Xu CS et al 2017 Enhanced FIB-SEM systems for large-volume 3D imaging. eLife 6, 1–36. (10.7554/eLife.25916) PMC5476429 28500755

[121] Xu CS, Pang S, Hayworth KJ, Hess HF 2019 Enabling FIB-SEM systems for large volume connectomics and cell biology. *bioRxiv* **C**, 852863 (10.1101/852863)

[122] Scheffer LK et al 2020 A connectome and analysis of the adult *Drosophila* central brain. *bioRxiv* 030213 (10.1101/2020.04.07.030213)

[123] Baena V, Terasaki M 2019 Three-dimensional organization of transzonal projections and other cytoplasmic extensions in the mouse ovarian follicle. Sci. Rep. 9, 1–13. (10.1038/s41598-018-37766-2) 30718581 PMC6362238

[124] So C, Seres KB, Steyer AM, Mönnich E, Clift D, Pejkovska A, Möbius W, Schuh M 2019 A liquid-like spindle domain promotes acentrosomal spindle assembly in mammalian oocytes. Science 364, eaat9557 (10.1126/science.aat9557) 31249032 PMC6629549

[125] Schertel A, Snaidero N, Han HM, Ruhwedel T, Laue M, Grabenbauer M, Möbius W 2013 Cryo FIB-SEM: volume imaging of cellular ultrastructure in native frozen specimens. J. Struct. Biol. 184, 355–360. (10.1016/j.jsb.2013.09.024) 24121039

[126] Hörning M, Schertel A, Schneider R, Lemloh ML, Schweikert MR, Weiss IM 2020 Mineralized scale patterns on the cell periphery of the chrysophyte Mallomonas determined by comparative 3D Cryo-FIB SEM data processing. J. Struct. Biol. 209, 107403 (10.1016/j.jsb.2019.10.005) 31614182

[127] Zhu Y, Sun D, Schertel A, Ning J, Fu X, Gwo PP, Watson AM, Zhang P 2020 Serial cryoFIB/SEM reveals profound cytoarchitectural disruptions caused by a pathogenic mutation in Leigh syndrome patient cells. *bioRxiv* (10.1101/2020.05.28.121111)

[128] Mühleip AW, Dewar CE, Schnaufer A, Kühlbrandt W, Davies KM 2017 In situ structure of trypanosomal ATP synthase dimer reveals a unique arrangement of catalytic subunits. Proc. Natl Acad. Sci. USA 114, 992–997. (10.1073/pnas.1612386114) 28096380 PMC5293049

[129] Kremer A et al 2015 Developing 3D SEM in a broad biological context. J. Microsc. 259, 80–96. (10.1111/jmi.12211) 25623622 PMC4670703

[130] Gorelick S, Buckley G, Gervinskas G, Johnson TK, Handley A, Caggiano MP, Whisstock JC, Pocock R, de Marco A. 2019 PIE-scope, integrated cryo-correlative light and FIB/SEM microscopy. eLife 8, 1–15. (10.7554/eLife.45919) PMC6609333 31259689

[131] Moser F, Prazák V, Mordhorst V, Andrade DM, Baker LA, Hagen C, Grünewald K, Kaufmann R 2019 Cryo-SOFI enabling low-dose super-resolution correlative light and electron cryo-microscopy. Proc. Natl Acad. Sci. USA 116, 4804–4809. (10.1073/pnas.1810690116) 30808803 PMC6421404

[132] Wu G et al 2020 Multi-scale 3D cryo-correlative microscopy for vitrified cells. *bioRxiv*.

[133] Hoffman DP et al 2020 Correlative three-dimensional super-resolution and block-face electron microscopy of whole vitreously frozen cells. Science 367, eaaz5357 (10.1126/science.aaz5357) 31949053 PMC7339343

[134] Hirose M et al 2020 Acrosin is essential for sperm penetration through the zona pellucida in hamsters. Proc. Natl Acad. Sci. USA 117, 2513–2518. (10.1073/pnas.1917595117) 31964830 PMC7007544

[135] Porter KR, Claude A, Fullam EF 1945 A study of tissue culture cells by electron microscopy: methods and preliminary observations. J. Exp. Med. 81, 233–246. (10.1084/jem.81.3.233) 19871454 PMC2135493

[136] Palade GE 1955 A small particulate component of the cytoplasm. J. Biophys. Biochem. Cytol. 1, 59–68. (10.1083/jcb.1.1.59) 14381428 PMC2223592

[137] Palade GE 1955 Studies on the endoplasmic reticulum. J. Biophys. Biochem. Cytol. 1, 567–582. (10.1083/jcb.1.6.567) 13278367 PMC2223828

[138] Palade GE 1953 An electron microscope study of the mitochondrial structure. J. Histochem. Cytochem. 1, 188–211. (10.1177/1.4.188) 13069686

[139] Comizzoli P, Holt WV 2019 Breakthroughs and new horizons in reproductive biology of rare and endangered animal species. Biol. Reprod. 101, 514–525. (10.1093/biolre/ioz031) 30772911

